# Supplementary material for: 6-Oxofurostane and (iso)Spirostane Types of Saponins in Smilax sieboldii: UHPLC-QToF-MS/MS and GNPS-Molecular Networking Approach for the Rapid Dereplication and Biodistribution of Specialized Metabolites
Source: Int J Mol Sci. 2023 Jul 14;24(14):11487. doi: 10.3390/ijms241411487 (PMC10380369; doi:10.3390/ijms241411487)
Supplement: Supplementary file 1 [file ijms-24-11487-s001.zip › ijms-2495807-supplementary.pdf]

International Journal of *Molecular Sciences*

Supplementary Materials

6-Oxofurostane and (iso)Spirostane Types of Saponins in *Smilax sieboldii*: UHPLC-QToF-MS/MS and GNPS-Molecular Networking Approach for the Rapid Dereplication and Biodistribution of Specialized Metabolites

Bharathi Avula [1,*,†], Ji-Yeong Bae [1,†,‡], Jongmin Ahn [1,§], Kumar Katragunta [1], Yan-Hong Wang [1], Mei Wang [1,‖], Yongsoo Kwon [1,¶], Ikhlas A. Khan [1,2] and Amar G. Chittiboyina [1,*]

[1] National Center for Natural Products Research, School of Pharmacy, University of Mississippi, University, MS 38677, USA; jybae@jejunu.ac.kr (J.-Y.B.); jmahn@kribb.re.kr (J.A.); kkatragu@olemiss.edu (K.K.); wangyh@olemiss.edu (Y.-H.W.); meiwang@olemiss.edu (M.W.); yskwon@kangwon.ac.kr (Y.K.); ikhan@olemiss.edu (I.A.K.)
[2] Division of Pharmacognosy, Department of BioMolecular Sciences, School of Pharmacy, University of Mississippi, University, MS 38677, USA
* Correspondence: bavula@olemiss.edu (B.A.); amar@olemiss.edu (A.G.C.)
† These authors contributed equally to this work.
‡ Current address: College of Pharmacy, Jeju Research Institute of Pharmaceutical Sciences and Interdisciplinary Graduate Program in Advanced Convergence Technology & Science, Jeju National University, Jeju 63243, Republic of Korea.
§ Current address: Natural Medicine Research Center, Korea Research Institute of Bioscience and Biotechnology, Cheongju 28116, Republic of Korea.
‖ Current address: Natural Products Utilization Research Unit, Agricultural Research Service, United States Department of Agriculture, University, MS 38677, USA.
¶ Current address: College of Pharmacy, Kangwon National University, Chuncheon 24341, Republic of Korea.

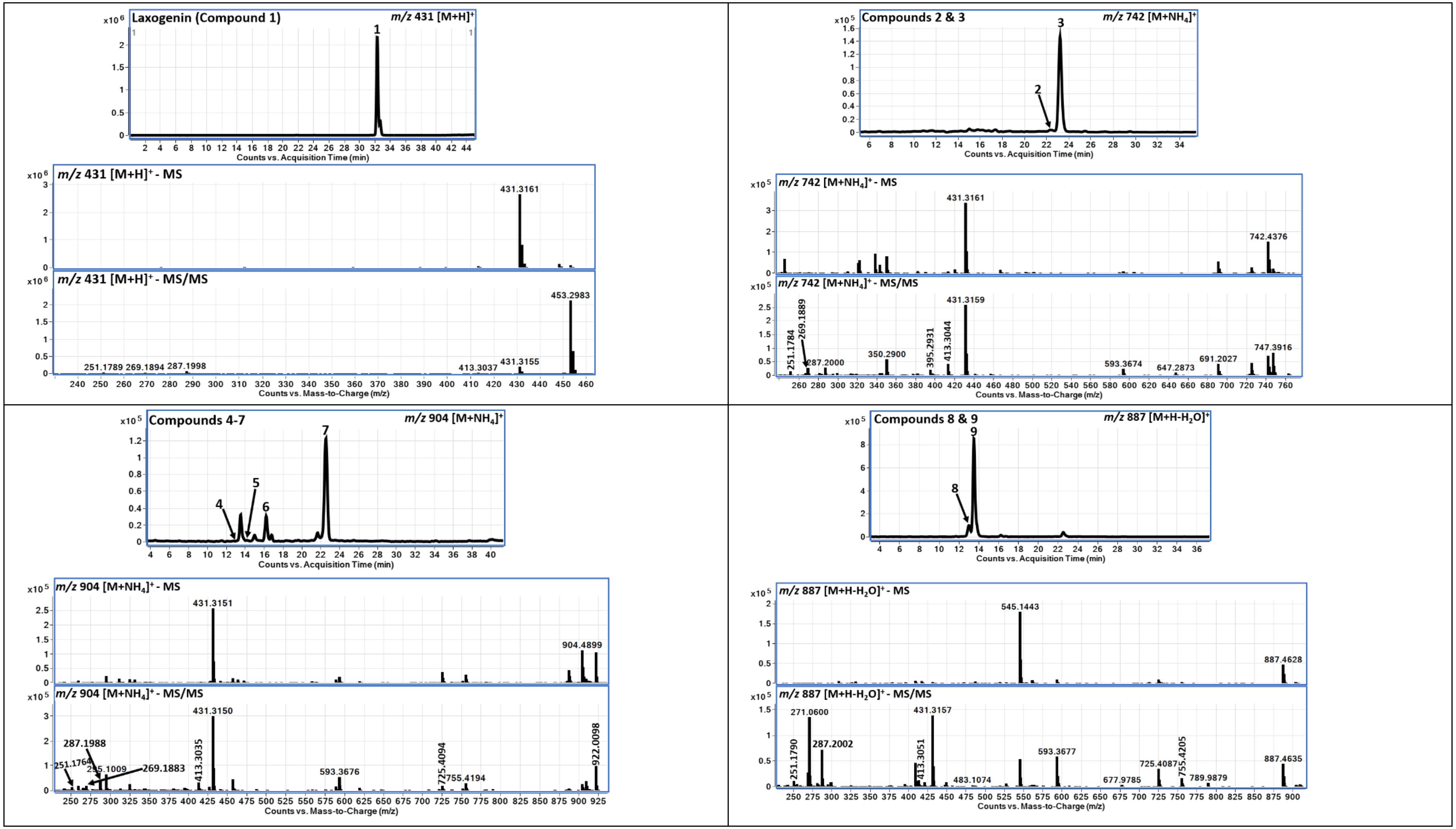

Figure S1. HRMS spectra for compounds (**1-41**) in *S. sieboldii* (continued)

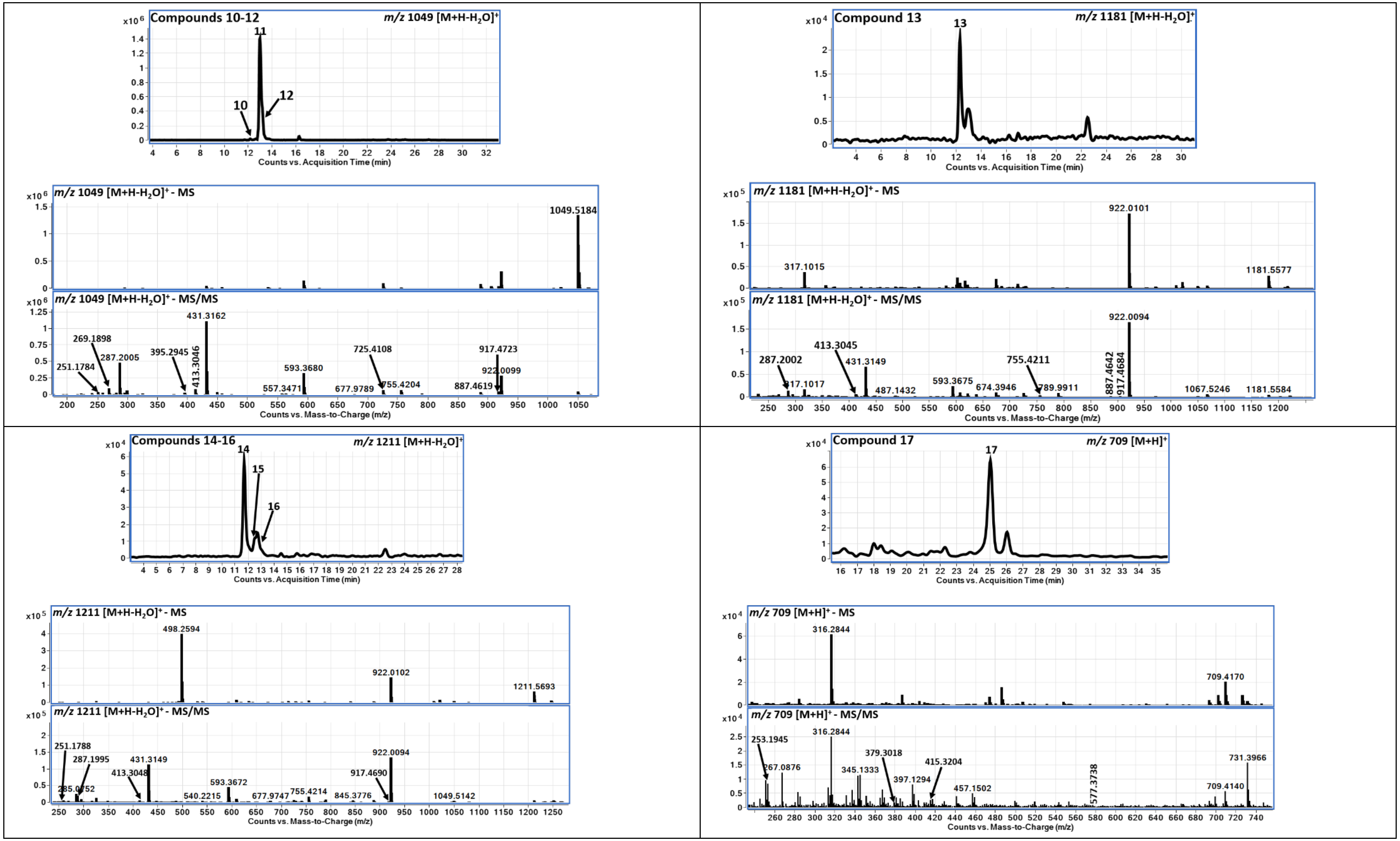
Compounds 10-12
m/z 1049 [M+H-H2O]+
Counts vs. Acquisition Time (min)
m/z 1049 [M+H-H2O]+ - MS
1049.5184
m/z 1049 [M+H-H2O]+ - MS/MS
431.3162
269.1898
287.2005
395.2945
413.3046
251.1784
593.3680
557.3471
677.9789
725.4108
755.4204
917.4723
922.0099
887.4619
Counts vs. Mass-to-Charge (m/z)
Compound 13
m/z 1181 [M+H-H2O]+
m/z 1181 [M+H-H2O]+ - MS
922.0101
317.1015
1181.5577
m/z 1181 [M+H-H2O]+ - MS/MS
922.0094
413.3045
287.2002
431.3149
317.1017
487.1432
593.3675
674.3946
755.4211
789.9911
887.4642
917.4684
1067.5246
1181.5584
Compounds 14-16
m/z 1211 [M+H-H2O]+
m/z 1211 [M+H-H2O]+ - MS
498.2594
922.0102
1211.5693
m/z 1211 [M+H-H2O]+ - MS/MS
251.1788
287.1995
431.3149
413.3048
285.0952
593.3672
540.2215
677.9747
755.4214
845.3776
917.4690
922.0094
1049.5142
Compound 17
m/z 709 [M+H]+
m/z 709 [M+H]+ - MS
316.2844
709.4170
m/z 709 [M+H]+ - MS/MS
253.1945
316.2844
267.0876
345.1333
379.3018
397.1294
415.3204
457.1502
577.3738
731.3966
709.4140

Figure S1. HRMS spectra for compounds (**1-41**) in *S. sieboldii* (continued)

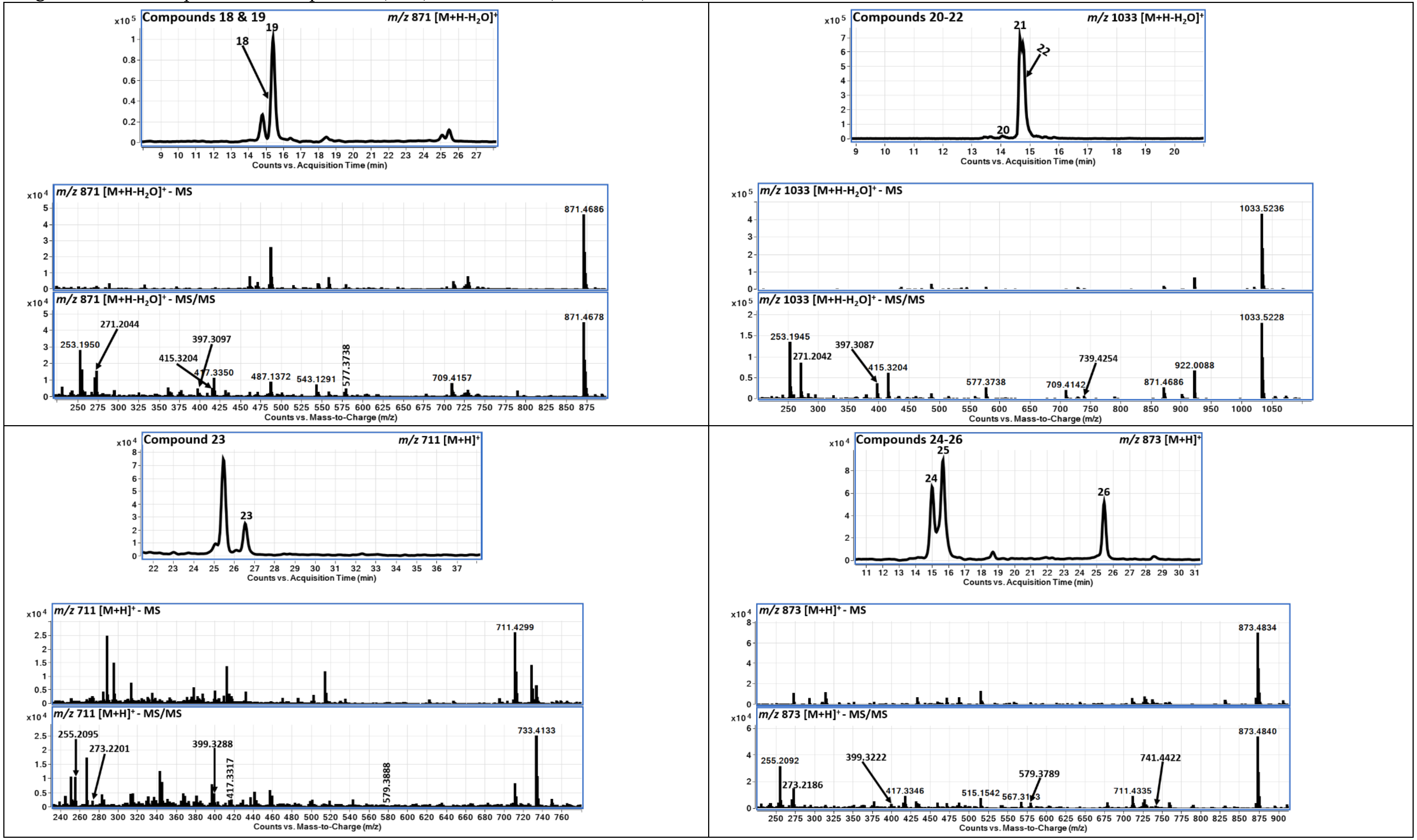

Figure S1. HRMS spectra for compounds (**1-41**) in *S. sieboldii* (continued)

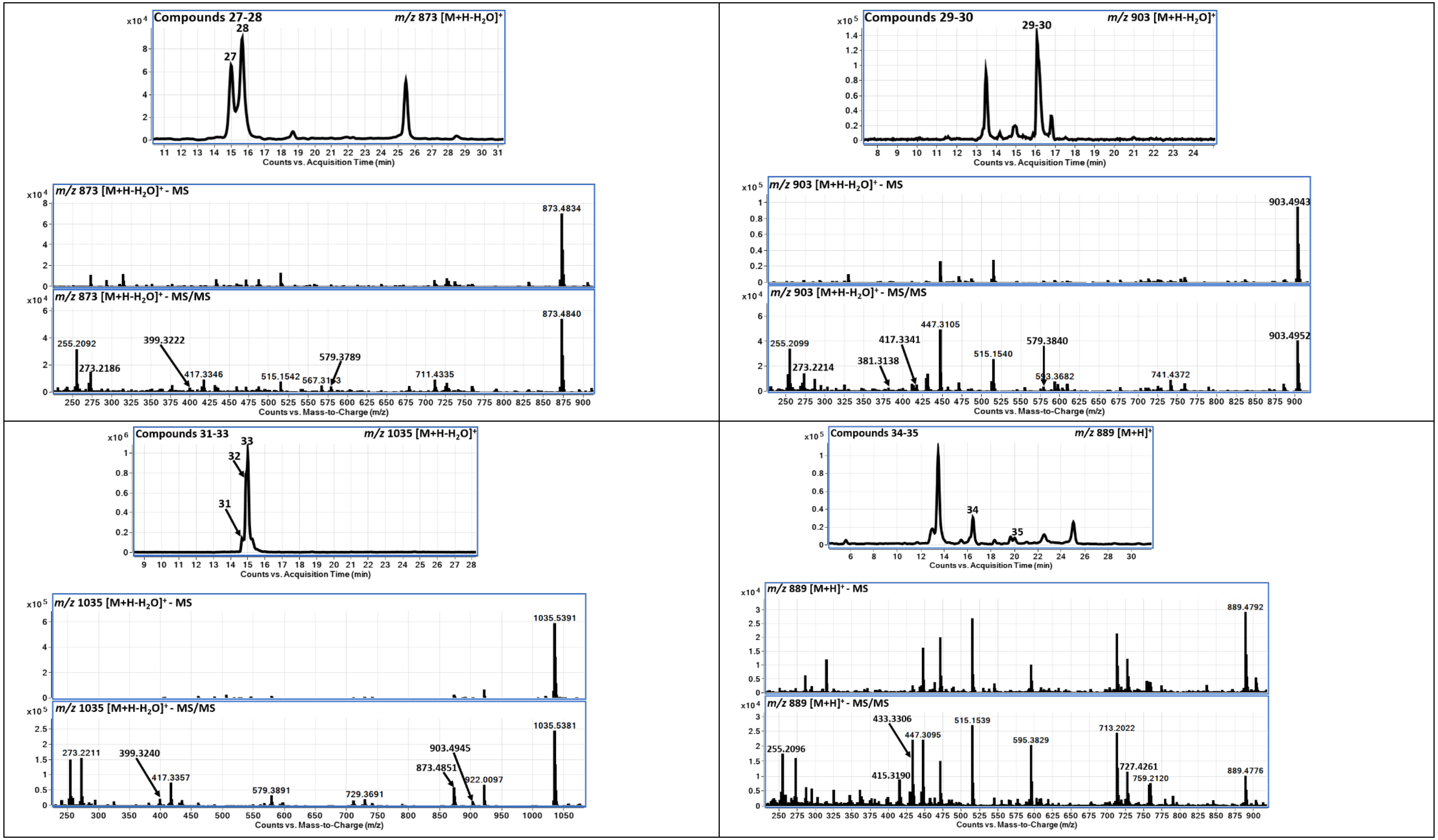

Figure S1. HRMS spectra for compounds (**1-41**) in *S. sieboldii* (continued)

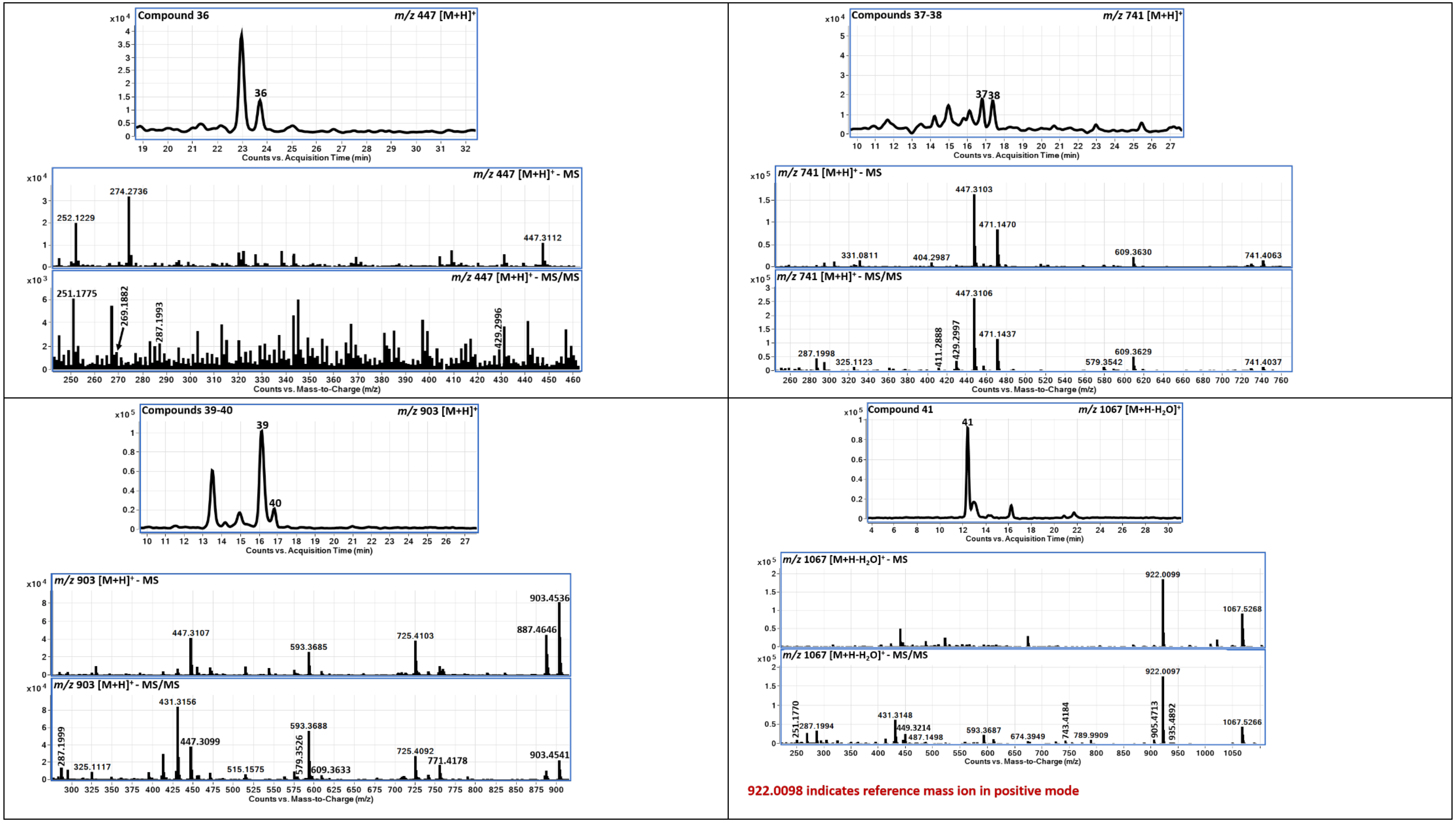

Figure S1. HRMS spectra for compounds (**1-41**) in *S. sieboldii* (continued)

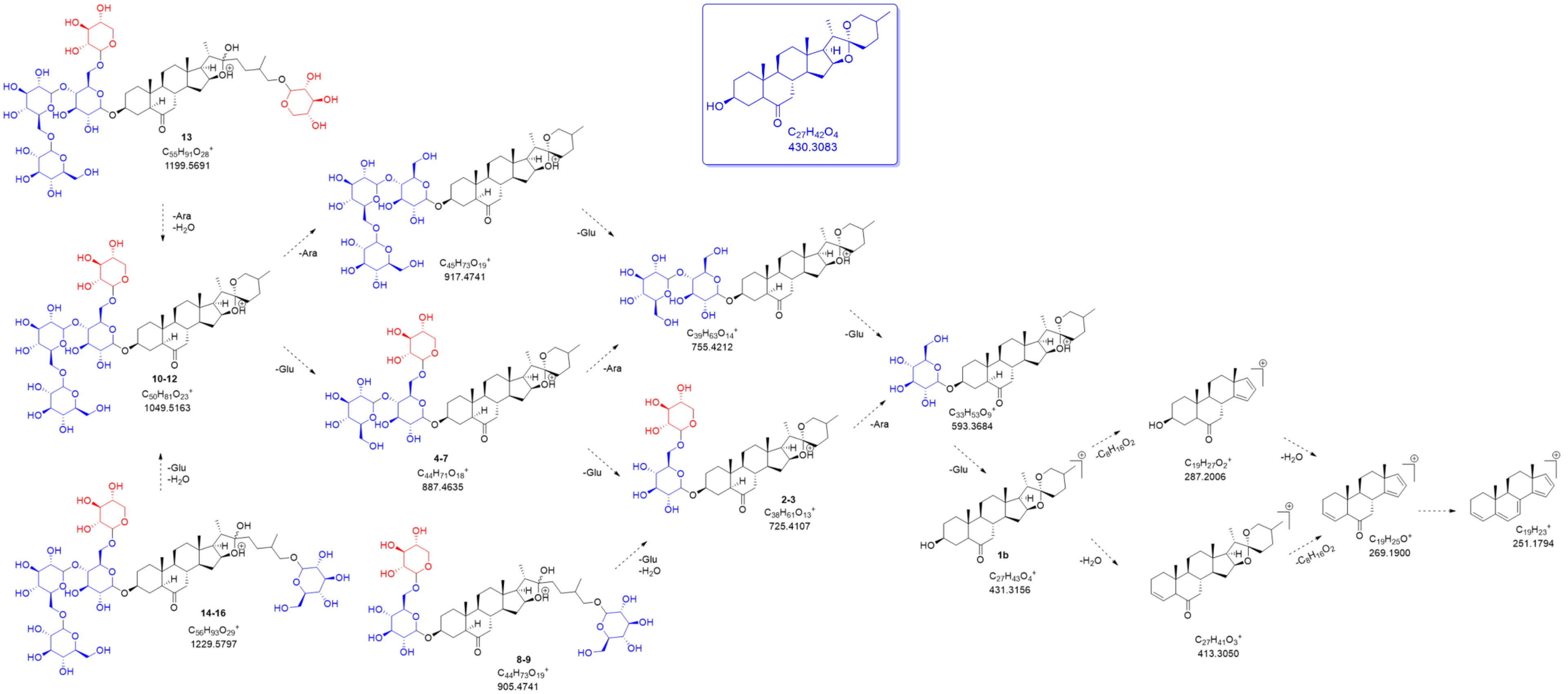

Figure S2. Mass fragmentation of laxogenin (1b)-based saponins (**2-16**) in *S. sieboldii*

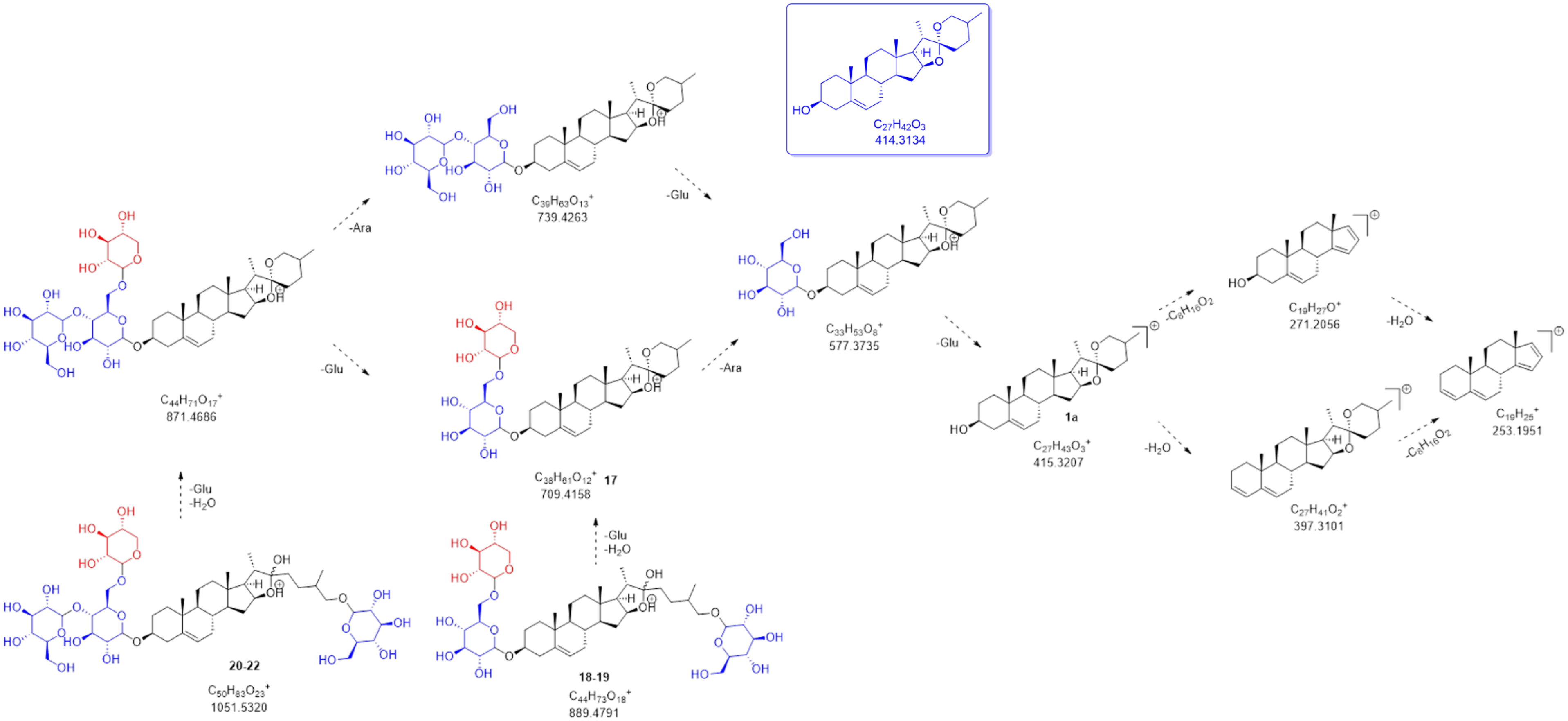

Figure S3. Mass fragmentation of diosgenin (1a)-based saponins (**17-22**) in *S. sieboldii*

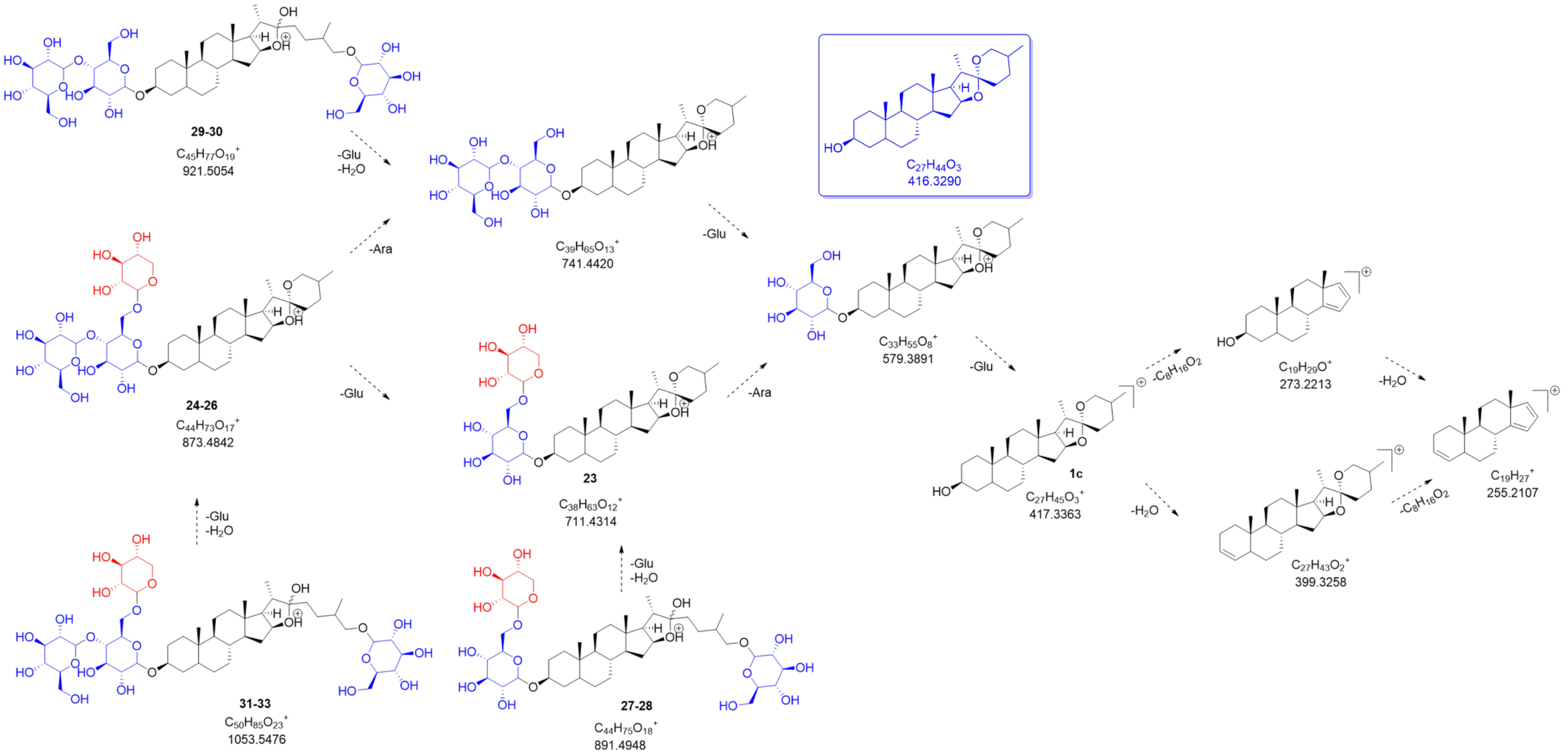

Figure S4. Mass fragmentation of tigogenin (1c)-based saponins (**23-33**) in *S. sieboldii*

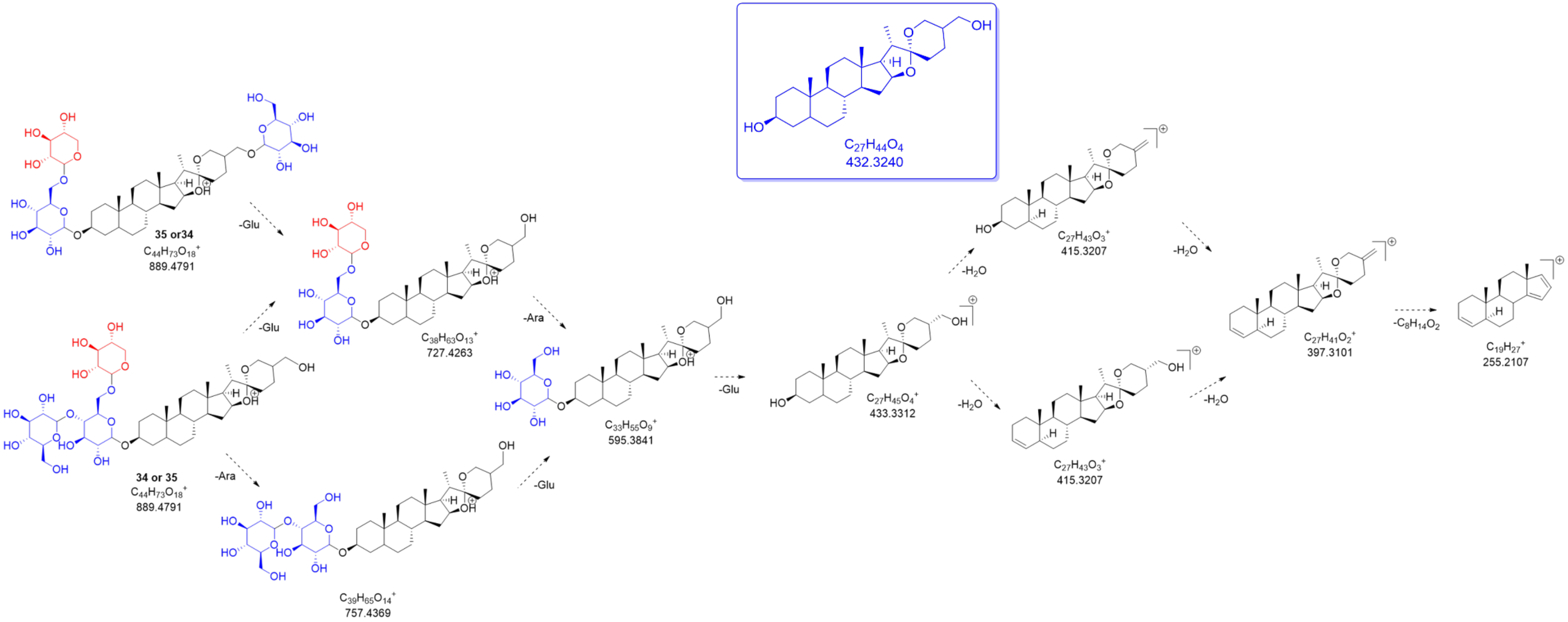

Figure S5. Mass fragmentation of 27-hydroxy (iso)spirostane-type saponins (**34** and **35**) in *S. sieboldii*

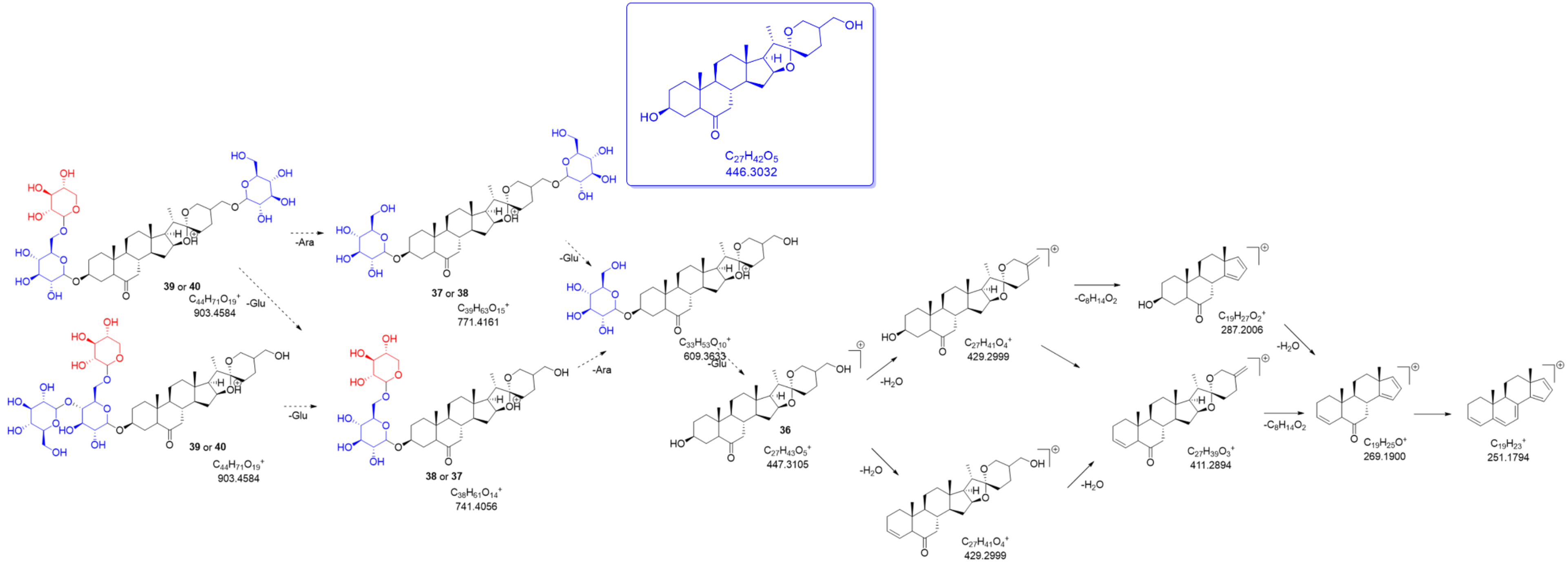

Figure S6. Mass fragmentation of 27-hydroxy (iso)spirostane-type saponins (**36-40**) in *S. sieboldii*

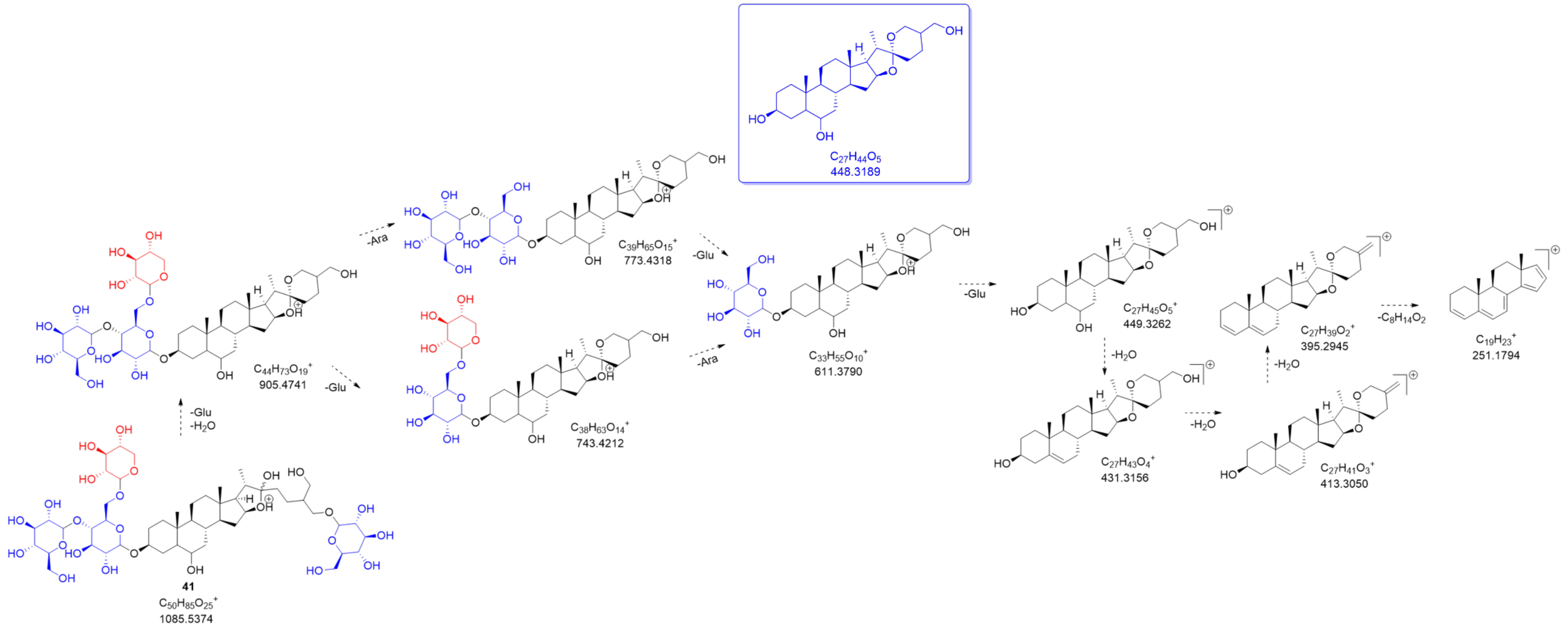

Figure S7. Mass fragmentation of 3,6,27-trihydroxy furostane-type saponin (**41**) in *S. sieboldii*
